# Systematic Review: Does Pre-Pubertal Spaying Reduce the Risk of Canine Mammary Tumours?

**DOI:** 10.3390/ani15030436

**Published:** 2025-02-04

**Authors:** Phillip Guirguis, David S. Beggs

**Affiliations:** Melbourne Veterinary School, University of Melbourne, 250 Princes Highway, Werribee, VIC 3030, Australia

**Keywords:** age, benefits, cancer, canine, desexing, gonadectomy, mammary, risks, spaying, systematic review, tumour

## Abstract

Background: Ovariectomy and ovariohysterectomy (spaying) are two surgical procedures commonly performed on dogs. However, the optimum age at which to perform these procedures in dogs remains a contentious issue. Following work published in the 1960s, pre-pubertal spaying became popular largely on the basis that it reduced the risk of mammary cancer development. However, a systematic review published more than 10 years ago questioned this. The objective of this study was to examine the strength and direction of updated literature on the relationship between spaying age of dogs and mammary tumour development and to investigate whether the evidence has changed. Method: A systematic review was conducted based on the Preferred Reporting Items for Systematic Reviews and Meta-Analyses (PRISMA) guidelines. In March 2023, peer-reviewed articles were collected from three databases (CAB direct, Web of Science, and PubMed). Articles were excluded if they were not in English, not peer-reviewed, not relevant to PICO (Population, Intervention, Comparison and Outcome), or if the study investigated the effects of desexing on canines with mammary tumours already or of the prognosis and not development of mammary tumours. Results: Of 232 articles obtained from the initial database search, 13 articles were found to meet the inclusion criteria. Six out of the thirteen papers selected found no evidence of a protective effect of early spaying against mammary tumour development. Conclusion: This review suggests that further breed-specific studies with matched controls on the main risk factors would help address issues such as lack of appropriate statistical analysis, control of biases, and confounding risk factors. The benefits of early spaying should be balanced with other considerations about the optimal age for spaying dogs.

## Simple Summary

Spaying is a common surgical procedure conducted in the veterinary industry and can involve the removal of both the uterus and ovaries (ovariohysterectomy) or just the ovaries (ovariectomy) in dogs. One of the commonly reported benefits of conducting this procedure early in a dog’s life includes a reduction in the development risk of mammary tumours. The aim of the present study is to examine the existing literature to determine the strength and direction of evidence of a potential protective effect of early spaying/pre-pubertal spaying on mammary tumour development throughout a dog’s life. Thirteen papers were included for analysis based on the inclusion criteria and six of these papers found no evidence of a protective effect of early spaying against mammary tumour development. Further studies that account for confounding factors such as breed differences are required to be able to elucidate any potential protective effects of early/pre-pubertal spaying and whether this impacts mammary tumour development. The benefits of early/pre-pubertal spaying should be considered and balanced with other considerations about the optimal age for spaying dogs.

## 1. Introduction

Spaying, which involves the surgical removal of the ovaries and often the uterus, is one of the most common surgical procedures conducted in the Australian veterinary industry. Historically, spaying was used as a population control measure, preventing the birth of unplanned/unwanted puppies and therefore helping to reduce the numbers of stray dogs [1]. This is still practised in many parts of the world today. The additional benefits proposed also included preventing the development of reproductive tract-related diseases such as pyometra and mammary cancers [2,3].

Under physiological conditions, oestrogen stimulates the growth of mammary tissue, and its proliferative effect on the epithelium may contribute to the development of mammary tumours [4], whilst progestins stimulate a lobulo-alveolar development with hyperplasia of the myoepithelial and secretory cells. This occurs during every oestrous cycle and subsequently makes a dog more susceptible to carcinogenesis as its cumulative exposure to oestrogen and progesterone increases throughout its lifetime [4]. Since mammary cancer development risk is influenced by cumulative oestrogen exposure, factors that reduce this would likely help limit mammary tumour development. As such, prepubertal spaying, when compared to intact or late spaying, will reduce the exposure of mammary tissues to oestrogen by reducing the oestrogen produced as well as reducing the amount of mammary tissue exposed, thus potentially reducing cancer development.

Studies into the optimal age at which spaying should occur have yielded conflicting findings [5]. Historically, the age at which spaying was performed in Australia was between five and nine months of age [6]. The first major study that recommended pre-pubertal spaying as a preventive surgery against mammary cancer development was published in 1969 [3]. This resulted in a shift in thinking toward spaying at an earlier age. Nowadays, pre-pubertal desexing is a common practice in many animal shelters and private veterinary clinics.

Several risk factors for canine mammary tumours have been identified, and it is likely that a combination of external factors and host susceptibility play significant roles in disease development. The most reported important risk factors are age, breed, genetic predisposition, hormones, and diet [4].

A systematic review published 11 years ago examined the proposed association between age at spaying and a protective effect against mammary cancer development [7]. Beauvais et al. found that due to the risk of bias and limited peer-reviewed evidence, the relationship between spay age and the development of mammary tumours was judged to be weak. Comparing the outcomes of various studies examining spay age and mammary tumour development has proved difficult due to significant differences in methodology, study populations, and confounding variables. There have been several more studies published since then, warranting further investigation on the existing body of evidence.

In this review, we examine evidence regarding the timing of desexing and the development of mammary tumours in dogs, and whether the age at desexing is a useful tool in reducing the incidence of this disease.

## 2. Methods

### 2.1. Protocol

A systematic review following the Preferred Reporting Items for Systematic Reviews and Meta-Analyses (PRISMA) guidelines was conducted [8].

### 2.2. Eligibility Criteria and Study Selection

All references were imported to Endnote x9 (Clarivate, PA, US), and duplicates were deleted using the automatic function of Endnote x9. Duplicates were deleted based on matching title and author(s). Any duplicates that were not deleted by Endnote x9 were deleted manually by the primary author.

The remaining articles were screened by the primary author and any articles that did not fulfil the inclusion criteria (Table 1) were excluded. The full article texts were subsequently retrieved for the remaining papers. The papers were then screened by the primary author to eliminate any articles that did not fulfil the inclusion criteria.

## 3. Results

### 3.1. Study Selection

**Figure 1:**
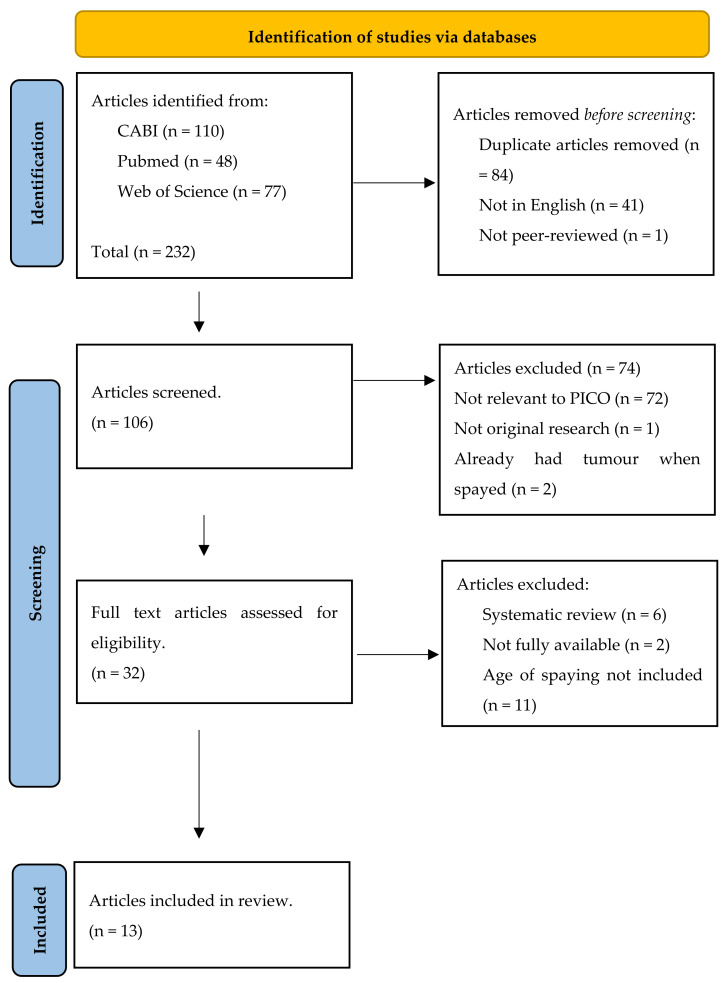
PRISMA flow chart for literature review on the optimum spaying age and its impact on mammary tumour development in dogs. PRISMA = Preferred Reporting Items for Systematic reviews and Meta-analysis; CABI = Commonwealth Agricultural Bureau International; PICO = Population, Intervention, Comparison and Outcome.

The initial search yielded 232 articles from the three databases. After the removal of duplicates and screening, 13 remained for analysis (Figure 1).

### 3.2. Study Characteristics

Beaudu-Lange et al. examined the clinical records of 599 dogs from a veterinary clinic in France and found that 160 dogs had mammary tumours. Dogs were classified as early spayed (spayed before 2 years old), late spayed (spayed after 2 years old), or entire (not spayed). Of these 160 dogs, 2 were early spayed, 13 were late spayed, and 145 dogs were intact. Compared to intact and late spayed, early spayed dogs were at a significantly lower risk of developing mammary tumours.

Gedon et al. examined the clinical records of 625 dogs diagnosed with mammary tumours from a veterinary clinic in Germany. In this study, 43 of the 123 neutered dogs had their age of neutering recorded. Of these 43 dogs, 2 were spayed at less than 2 years old, 2 were spayed at less than 3 years old, and 39 were spayed between 4 years and 14 years old. Five hundred and two dogs had mammary tumour(s) and were intact. Gedon et al. concluded that spaying later in life still reduces the risk of mammary tumour development when compared to entire dogs.

Hart et al. (2014) examined the clinical records of 1500 Labrador retrievers and 1015 Golden retrievers from the Veterinary Teaching Hospital, University of California-Davis. They found that 3.5% of Golden retrievers spayed after 2 years of age were diagnosed with mammary tumours. No mammary tumours were detected in entire dogs or those spayed before 2 years of age. Further, 2% of Labrador retrievers that were spayed older than 2 years were diagnosed with mammary tumours. In addition, 1.4% of the entire Labrador retriever population examined developed mammary tumours.

Santos et al. screened 386 dogs for mammary tumours in Brazil between 2015 and 2017. They found that 23.6% of the 386 dogs had mammary tumours. Dogs admitted for spay after the third oestrous cycle had a higher frequency of mammary tumours (27.6%) compared to dogs submitted before the third oestrous cycle (9.4%). Entire dogs were 9.34 times more likely to have a mammary tumour compared to those spayed.

Schneider et al. (1969) examined the clinical records of 93 dogs with histologically confirmed mammary tumours between July 1963 and June 1965 at Alameda County, USA. One dog developed mammary tumours after being spayed before any oestrous cycle. Three dogs developed mammary tumours after being spayed after one oestrous cycle. Twenty dogs developed mammary tumours after being spayed after two or more oestrous cycles. Scheinder et al. (1969) concluded that dogs spayed before any oestrous cycle had 0.5% of the risk of developing mammary tumours when compared to intact dogs. Dogs that were spayed after only one oestrous cycle had 8% of the risk of developing mammary tumours. Spaying before the age of 2.5 years had a significant sparing effect on the development of mammary tumours when compared to those spayed after 2.5 years old.

Sonnenschein et al. examined the records of 428 dogs from the Outpatient Diagnostic Service of the University of Pennsylvania between 1982 and 1983. The odds ratio for spaying at 1 year old, relative to intact, was 0.01 for cases when compared with cancer and non-cancer controls. Spaying between 1.1 and 2.5 years of age was protective.

Fidler and Brodey (1967) examined the clinical records of 161 dogs diagnosed with mammary tumours at the University of Pennsylvania Veterinary Hospital between 1963 and 1966. Of the 161 dogs, 21 dogs had been spayed. The specific age groups of spayed dogs were not reported; however, the authors report that age of spaying had correlated with the age at the time of mammary tumour diagnosis (r = 0.646, *p* < 0.01) and that dogs spayed at an older age were more likely to develop mammary tumours [9].

de la Riva et al. examined the clinical records of 759 Golden retrievers admitted to the Veterinary Teaching Hospital of the University of California-Davis. In this study, 172 dogs were spayed before 1 year and 70 dogs were spayed after 1 year. No mammary tumours were detected in the Golden retriever population.

Hart et al. (2016) examined the clinical records of 1170 German Shepherd dogs admitted to the Veterinary Teaching Hospital at the University of California-Davis. Of these dogs, 465 were female and 293 were neutered. There was no diagnosis of mammary tumours in dogs spayed at less than 6 months of age. Further, 1.11% of dogs spayed between 6 and 11 months were diagnosed with mammary tumours. Additionally, 2.7% of dogs spayed at 1 year old were diagnosed with mammary tumours, and 4.9% of dogs spayed between 2 and 8 years old were diagnosed with mammary tumours. Furthermore, 4.1% of intact dogs were diagnosed with mammary tumours. There was no statistical significance difference in the diagnosis rates of the German Shepherds.

Sorenmo et al. examined the clinical records of 137 dogs diagnosed with mammary tumours through the Surgical Pathology Service of the Veterinary Hospital of the University of Pennsylvania. Spay age was not specifically reported; however, the authors mentioned that dogs grouped by spay age were not statistically different regarding factors such as vascular invasion or number and size of nodules within mammary tumours. Dogs in the intact group were more likely to have an anaplastic tumour type.

Waters et al. analysed 242 responses to a questionnaire sent to Rottweiler owners who were members of specialty Rottweiler clubs. No dogs that were spayed at less than 2 years of age developed mammary tumours. The rate ratio of dogs spayed between 2.5 and 4.9 years old was 1, whilst the rate ratio of dogs spayed between 5 and 7.4 years old was 1.6. Dogs that were spayed older than 7.5 years old had a rate ratio of 1.9.

Zink et al. examined the responses to a questionnaire sent to Vizslas owners across multiple countries. Herein, 1360 were included in this study, and of these, 535 were intact. Eleven dogs had mammary tumours. Of these eleven dogs, ten dogs were spayed at an age greater than five years.

Else and Hannant examined the clinical records of 226 dogs. They found that 23 dogs had been spayed (10.2% of bitches in the survey). Of these twenty-three dogs, three had been spayed after two years old. The authors report that limited conclusions could be drawn regarding the influence of spaying age on mammary tumour development due to small group sizes and a lack of age-matched controls.

## 4. Discussion

The articles include evidence for the impact of spaying age on mammary tumour development in a variety of dog breeds and at different geographical locations/time periods. There were no studies that were considered to be conclusive. Studies that were deemed to have an adequate design were small studies that lacked power and control of confounding variables and presented risk factors that could significantly impact the conclusions made. Most studies in this systematic review are case controls. Due to their retrospective nature, case–control studies do not establish causation and instead present odds ratios—evidence for correlation between exposures and outcomes [10]. Various factors can predispose dogs to the development of canine mammary cancers. Therefore, whilst there may be correlation between early spaying and the development of mammary cancers, there are other factors, such as diet, weight, and age, which were not controlled or reported on in many studies examined.

### 4.1. Evidence for Early Spaying

Seven out of the thirteen papers provided evidence to support a protective effect of early spaying against the development of mammary neoplasms. These studies were inconsistent as to whether they grouped spaying by age or number of oestrous cycles experienced by the dog. Consequently, it was difficult to delineate between early spaying (spaying conducted after the bitch has experienced an oestrous cycle) and pre-pubertal spaying (spaying conducted before any oestrous cycle has occurred). The age at which bitches enter heat is breed-specific [11]. Smaller breeds typically experience their first heat earlier than larger breed dogs. Small breed dogs undergo their first heat at as early as 5–6 months, whilst larger dog breeds reach puberty as late as 18 months. Therefore, smaller breeds typically experience more oestrous cycles at an earlier age and are therefore exposed to higher levels of oestrogen and other mammary tissue-stimulating hormones before spaying. This increases the amount of breast tissue development and therefore could result in higher levels of mammary tumour development. Larger breed dogs are typically less likely to have experienced as many oestrous cycles prior to spaying due to the comparatively delayed onset of puberty.

Additionally, one study found that whilst spaying, at any time in life, reduced the risk of mammary tumour development, spayed bitches had mammary neoplasms of more malignant subtypes [12]. In this study, tumours considered to be of advanced malignancy were twice as common in spayed patients when compared to intact patients. For example, 15% of mammary tumours in spayed dogs were anaplastic carcinomas, compared to 7.6% in intact dogs (OR: 2.1234, 95%CI: 1.3782–3.2717; *p* = 0.0006). This suggests that early spaying may be less protective against the development of mammary tumours with a poorer prognosis. A possible explanation for this could be a reduction in oestrogen-responsive tumours but no reduction in non-oestrogen responsive tumours. Therefore, the overall tumour rate is lower in spayed females, but there are more tumours of higher-grade malignancy. Sorenmo et al. supports this, as they found that dogs that were spayed more than two years prior to tumour removal were more likely to have oestrogen-receptor negative tumours.

### 4.2. No Evidence for Early Spaying

Six out of the thirteen papers found no evidence to support a protective effect of early spaying for the prevention of mammary neoplasm development. One study on German Shepherds found that only 4% of intact bitches were diagnosed with mammary tumours, which suggests that mammary cancer is not a specific problem in this breed [13]. Similarly, another study found low rates of mammary neoplasm development in Labrador Retrievers and Golden Retrievers. This suggests specific genetic breed-line differences in mammary neoplasm predisposition.

Additionally, these studies found that whilst mammary neoplasm development was largely unaffected by early spaying, the incidence of other diseases increased. For example, one study found that the incidence of cranial cruciate ligament tear and rupture increased in spayed Golden Retriever and Labrador Retriever bitches compared to intact bitches of the same breed [14]. Additionally, hip dysplasia was elevated in early spayed Labrador Retriever bitches when compared to those intact. This suggests that whilst early spaying may be protective against the development of mammary neoplasms, it may simultaneously increase the prevalence of other diseases that select breeds have a higher genetic predisposition to developing.

### 4.3. Confounding Variables

Various confounding risk factors that are thought to contribute to the development of mammary tumours in dogs have been previously identified and discussed [3,4]. All the included literature fails to control at least one or more of these factors, and this therefore makes it difficult to draw meaningful conclusions.

#### 4.3.1. Age

Canine mammary tumours have a median age of development between 8 and 10 years of age. The age of a bitch is an important confounding variable, and one study in this review found that the risk of breast cancer increases by 1.625 times annually [15]. Two studies conducted by Hart et al. excluded clinical records of dogs over 9 years old, which could have influenced the relatively low level of mammary tumours detected in the cohorts examined, affecting the internal validity of each study [13,14]. Another study failed to match ages between control and exposure groups, contributing to confounding [12].

#### 4.3.2. Breed and Genetic Predisposition

Purebred dogs were significantly overrepresented among cases of canine mammary tumours [16,17]. Breeds such as Poodles, English Springer Spaniels, Cocker Spaniels, German Shepherds, Yorkshire Terriers, and Dachshunds have been reported to have an increased risk of mammary tumour development. Similarly to age, some studies failed to appropriately match breeds between spayed and non-spayed groups [18,19]. Else and Hannant (1979) had such few representatives for certain breeds that meaningful assessment could not be conducted [20]. Another study categorised bitches based on breed size rather than the specific breed itself [21]. Other studies in this review explicitly examined one breed [13,22,23,24]. The lack of consistency in reporting breeds limits the external validity and prevents appropriate comparison.

#### 4.3.3. Hormones and Growth Factors

Ovarian steroids are responsible for the growth of mammary tissue. Because of their proliferative effect on mammary tissue, they may provide optimal conditions for neoplastic development. Due to the cyclic nature of the oestrous cycle, with every cycle the bitch becomes more susceptible to cancer development. Similarly to breed, many studies are inconsistent in the ages at which spaying was reported. For example, Fidler and Brodey (1967) [9] reported spaying based on age in years. In comparison, Schneider et al. (1969) [16] reported spaying age based on the number of oestrous cycles experienced.

#### 4.3.4. Diet

Obesity and a high-fat diet have been linked to an increased risk of mammary cancer development in dogs. Obesity and dietary fat content are potential confounding variables that may influence the development of mammary tumour development when not appropriately controlled. Many studies did not report on the diet of the bitches in their findings. Sonnenschein et al. (1991) [21] provided a questionnaire to owners to collect data on weight and diet. However, as this was based on owner memory, it introduced recall bias into the study. Similarly, Santos et al. (2020) [15] considered diet; however, there was no elaboration on fat content.

#### 4.3.5. Pre-Pubertal Spaying

None of the included studies mention and detail a thorough examination in order to confirm the pre-pubertal status of dogs where necessary. The distinction between early spaying (spaying after the onset of puberty but relatively early within the entirety of a dog’s life) and pre-pubertal spaying (spaying conducted prior to any oestrous cycles) is an important one. As mentioned previously, the cumulative exposure of gonadal hormones, namely oestrogen and progesterone, has a significant and proliferative effect on the development of mammary tissue and thus the potential for mammary tumour development. As a consequence of this, it would be reasonable to assume that differences between these spaying age groups would be present; however, based on current published studies, this is unclear.

#### 4.3.6. Direction and Strength of Evidence

There was no clear direction to the evidence due to differences in study design, inclusion criteria, and study population, as well as confounding variables, and the use of appropriate measures of association.

The strength of the evidence in the studies is weak. Many studies, whilst stating significance, did not use appropriate measures of association. As most of the studies were case–controls, the expected measure of association was an odds ratio. However, the studies were inconsistent as to whether an odds ratio was included. For example, Schneider et al. (1969) [16] used a relative risk calculation, whilst Fidler and Brodey (1967) [9] and Else and Else (1979) [20] used a chi-square test. In addition, Schneider et al. (1969) [16] lacked clarity on whether the random selection of cases had occurred, suggesting selection bias. Bias combined with inappropriate statistical calculations meant that the overall strength of evidence, whilst suggestive of a potential protective effect of early spaying, was weak.

### 4.4. Limitations of Studies

Many of the included publications suffer from similar biases. These include that the dogs included in the studies were of different ages and therefore had undergone a different number of oestrous cycles at the time of spaying. This would also be the case with these dogs before mammary tumour diagnosis. Due to the impact of the cumulative exposure of oestrogen and progesterone on the development and growth of mammary tissue, these factors could have a profound impact on the relationship between age of spay and mammary tumour development. This significantly impacts the comparability between groups and studies.

Due to the lack of breed-specific studies, the external validity of several included papers is limited. Whilst some papers provide evidence that the effects of spaying on mammary tumours can be significantly influenced by breed, the majority of studies are not breed-specific. This is because breed-specific vulnerabilities and certain diseases may not occur in other breeds. Therefore, the counselling given by veterinarians on when certain breeds should be spayed will be limited based on existing evidence.

Gedon et al. reported that the age of neutering was only available for 43 out of 123 dogs, and Fidler and Brodey report that only 21 out of 161 dogs included in the study were spayed. These small sample sizes are likely to be influenced by outliers, and findings based on these groups may not be representative of the whole population.

Additionally, several studies provided a questionnaire to the owners of the dogs selected [19,23,24]. The use of questionnaires introduces recall bias into these studies, as in one study, owners were reporting the disease conditions of dogs born as many as 16 years previously. Recall bias can increase or decrease the observed association between spaying and the development of mammary tumours [24].

Whilst some of the studies included in this review provide breed-specific conclusions, the inconsistent study methodologies (with varying and small sample sizes), inconsistent statistical analyses, and aforementioned biases make it difficult to derive meaningful conclusions and reduce strength of evidence available in the existing literature. Future research into a potential protective effect of spaying against mammary tumour development should look to involve larger sample sizes of individual breeds with control of the aforementioned confounding variables (such as diet). Future studies should also ensure that the dogs included have experienced no oestrous cycles (for pre-pubertal spaying) or a similar number of oestrous cycles (for early spaying).

A recent review, published after the present paper was completed [25], considered the ideal age at which dogs should be desexed in a much wider context, with a broad range of outcomes including orthopaedic disease, atopy, neoplasia, obesity, and urogenital disease. This study reported on only three papers from our study that specifically considered the relationship between pre-pubertal desexing and mammary carcinomas [3,18,22]; however, the conclusions reached with respect to mammary neoplasia were consistent with ours, noting the weaknesses in many previous studies, and concluding there was insufficient evidence regarding the optimal timing of desexing to provide evidence-based guidelines. Our discussion of the specific confounding variables related to mammary carcinomas, based on the 13 papers, complements the findings of this review.

## 5. Conclusions

Whilst there is evidence that early spaying could have a considerable sparing effect on the development of mammary tumours, the strength of the evidence supporting this is inconsistent. Several studies lack appropriate statistical analysis, control of biases, and confounding risk factors. Further breed-specific studies with matched controls on the main risk factors would help address these research gaps and evaluate the risks and benefits of pre-pubertal vs. late spaying, and whether some breeds may benefit from surgery. Whilst the prevention of mammary tumours is probably a benefit of early spaying, this needs to be balanced with other considerations about the optimal age for spaying dogs.

## Figures and Tables

**Table 1:** Search strategy for literature review on the optimum spaying age and its impact on mammary tumour development in dogs.

Databases	CABI	Web of Science	Pubmed
Date of Search	4 March 2023		
Dates included	1900—March 4th 2023		
Search input	(dog OR canine) AND (age) AND (spay* OR neuter* OR desex* OR ovariohysterectom* OR ovariectom* OR gonadect*) AND (mammary) AND (tumor* OR neoplas* OR growth OR cancer)		
PICO	Spayed dogs, spaying, different ages spaying conducted, development of mammary tumours.		
Inclusion criteria	Examines the impact of desexing age on development of mammary tumours in dogs		
Exclusion criteria	Not relevant to PICO question Not English Not peer-reviewed Not original research Abstract only Study investigates the effects of desexing on canines with mammary tumours already. Study investigates prognosis and not development of mammary tumours		

CABI = Commonwealth Agricultural Bureau International. PICO = Population, Intervention, Comparison and Outcome.

## Data Availability

No new data were created or analysed in this study. Data sharing is not applicable to this study.

## References

[1] Hart L.A., Hart B.L. (2021). An Ancient Practice but a New Paradigm: Personal Choice for the Age to Spay or Neuter a Dog. Front. Vet. Sci..

[2] McCallin A.J., Hough V.A., Kreisler R.E. (2021). Pyometra Management Practices in the High Quality, High Volume Spay-Neuter Environment. Top. Companion Anim. Med..

[3] Schneider R., Dorn C.R., Taylor D.O.N. (1969). Factors influencing canine mammary cancer development and postsurgical survival. J. Natl. Cancer Inst..

[4] Sleeckx N., de Rooster H., Veldhuis Kroeze E., Van Ginneken C., Van Brantegem L. (2011). Canine Mammary Tumours, an Overview. Reprod. Domest. Anim..

[5] Howe L.M. (2015). Current perspectives on the optimal age to spay/castrate dogs and cats. Vet. Med.-Res. Rep..

[6] Orr B., Jones B. (2019). A Survey of Veterinarian Attitudes Toward Prepubertal Desexing of Dogs and Cats in the Australian Capital Territory. Front. Vet. Sci..

[7] Beauvais W., Cardwell J.M., Brodbelt D.C. (2012). The effect of neutering on the risk of mammary tumours in dogs—A systematic review. J. Small Anim. Pract..

[8] Page M.J., McKenzie J.E., Bossuyt P.M., Boutron I., Hoffmann T.C., Mulrow C.D., Shamseer L., Tetzlaff J.M., Akl E.A., Brennan S.E. (2021). The PRISMA 2020 statement: An updated guideline for reporting systematic reviews. BMJ.

[9] Fidler I.J., Brodey R.S. (1967). The biological behavior of canine mammary neoplasms. J. Am. Vet. Med. Assoc..

[10] Mann C.J. (2003). Observational research methods. Research design II: Cohort, cross sectional, and case-control studies. Emerg. Med. J..

[11] Gobello C. (2014). Prepubertal and Pubertal Canine Reproductive Studies: Conflicting Aspects. Reprod. Domest. Anim..

[12] Gedon J., Wehrend A., Kessler M. (2022). Ovariectomy reduces the risk of tumour development and influences the histologic continuum in canine mammary tumours. Vet. Comp. Oncol..

[13] Hart B.L., Hart L.A., Thigpen A.P., Willits N.H. (2016). Neutering of German Shepherd Dogs: Associated joint disorders, cancers and urinary incontinence. Vet. Med. Sci..

[14] Hart B.L., Hart L.A., Thigpen A.P., Willits N.H. (2014). Long-Term Health Effects of Neutering Dogs: Comparison of Labrador Retrievers with Golden Retrievers. PLoS ONE.

[15] Santos T.R., Castro J.R., Andrade J.C., Silva A.C.R., Silva G.M.F., Ferreira F.A., Headley S.A., Saut J.P.E. (2020). Risk factors associated with mammary tumors in female dogs. Pesqui. Vet. Bras..

[16] Schneider R. (1970). Comparison of age, sex, and incidence rates in human and canine breast cancer. Cancer.

[17] Dorn C.R., Schneider R. (1976). Inbreeding and canine mammary cancer: A retrospective study. J. Natl. Cancer Inst..

[18] Beaudu-Lange C., Larrat S., Lange E., Lecoq K., Nguyen F. (2021). Prevalence of Reproductive Disorders including Mammary Tumors and Associated Mortality in Female Dogs. Vet. Sci..

[19] Sorenmo K.U., Shofer F.S., Goldschmidt M.H. (2000). Effect of spaying and timing of spaying on survival of dogs with mammary carcinoma. J. Vet. Intern. Med..

[20] Else R.W., Hannant D. (1979). Some epidemiological aspects of mammary neoplasia in the bitch. Vet. Rec..

[21] Sonnenschein E.G., Glickman L.T., Goldschmidt M.H., McKee L.J. (1991). Body conformation, diet, and risk of breast cancer in pet dogs: A case-control study. Am. J. Epidemiol..

[22] de la Riva G.T., Hart B.L., Farver T.B., Oberbauer A.M., Messam L.L.M., Willits N., Hart L.A. (2013). Neutering Dogs: Effects on Joint Disorders and Cancers in Golden Retrievers. PLoS ONE.

[23] Waters D.J., Kengeri S.S., Maras A.H., Suckow C.L., Chiang E.C. (2017). Life course analysis of the impact of mammary cancer and pyometra on age-anchored life expectancy in female Rottweilers: Implications for envisioning ovary conservation as a strategy to promote healthy longevity in pet dogs. Vet. J..

[24] Zink M.C., Farhoody P., Elser S.E., Ruffini L.D., Gibbons T.A., Rieger R.H. (2014). Evaluation of the risk and age of onset of cancer and behavioral disorders in gonadectomized Vizslas. J. Am. Vet. Med. Assoc..

[25] Moxon R., England G.C.W., Payne R., Corr S.A., Freeman S.L. (2024). Effect of neutering timing in relation to puberty on health in the female dog–a scoping review. PLoS ONE.

